# Incorporation of imidazolium chitosan derivative yields scaffolds with enhanced antioxidant, antimicrobial and immunomodulatory properties

**DOI:** 10.1093/rb/rbag077

**Published:** 2026-04-20

**Authors:** Carolina Muñoz-Núñez, Arantza Barco-Martín, Ketaki Deshpande, Dominik S Schmidt, Lola González-García, Sara Trujillo, Alexandra Muñoz-Bonilla, Marta Fernández-García

**Affiliations:** Instituto de Ciencia y Tecnología de Polímeros (ICTP-CSIC), Madrid 28006, Spain; Interdisciplinary Platform for Sustainable Plastics Towards a Circular Economy-Spanish National Research Council (SusPlast-CSIC), Madrid 28006, Spain; Facultad de Ciencias Químicas, Universidad Complutense de Madrid, Madrid 28040, Spain; Instituto de Ciencia y Tecnología de Polímeros (ICTP-CSIC), Madrid 28006, Spain; INM-Leibniz Institute for New Materials, Saarbrucken 66123, Germany; INM-Leibniz Institute for New Materials, Saarbrucken 66123, Germany; INM-Leibniz Institute for New Materials, Saarbrucken 66123, Germany; Department of Materials Science and Engineering, Saarland University, Saarbrucken 66123, Germany; INM-Leibniz Institute for New Materials, Saarbrucken 66123, Germany; Instituto de Ciencia y Tecnología de Polímeros (ICTP-CSIC), Madrid 28006, Spain; Interdisciplinary Platform for Sustainable Plastics Towards a Circular Economy-Spanish National Research Council (SusPlast-CSIC), Madrid 28006, Spain; Instituto de Ciencia y Tecnología de Polímeros (ICTP-CSIC), Madrid 28006, Spain; Interdisciplinary Platform for Sustainable Plastics Towards a Circular Economy-Spanish National Research Council (SusPlast-CSIC), Madrid 28006, Spain

**Keywords:** scaffolds, chitosan, antioxidant, antimicrobial, immune response

## Abstract

Developing biocompatible scaffolds with physicochemical properties suitable for regenerative medicine that also support cell adhesion and proliferation, while reducing local oxidative stress, exhibiting low immunogenicity and antimicrobial properties, represents a key objective in tissue engineering. Chitosan (CS), a biocompatible and biodegradable biopolymer, has attracted considerable attention in recent years as in tissue engineering. Herein, CS scaffolds were functionalized with bioactive antioxidant/antimicrobial molecules and reinforced with natural fillers to enhance these critical properties. The incorporation of a CS derivative containing 1-methylimidazole into CS-based scaffolds, and chitin nanowhiskers as reinforcement, was investigated. The resulting scaffolds exhibited an interconnected porous structure, facilitating nutrient diffusion and cell infiltration. Rheological analysis confirmed a predominantly soft and elastic behavior. Additionally, the antioxidant activity of the scaffolds was evaluated using the DPPH assay, while its antimicrobial effects were confirmed through bacterial inhibition tests. Fibroblast proliferation assays revealed an initial rapid growth phase followed by a stabilization. Immunological studies using macrophages demonstrated an initial activation of the NFκB transcription factor that did not result in the secretion of the pro-inflammatory cytokine IL-6, suggesting a transient macrophage activation. These findings highlight the potential of these CS-based scaffolds for biomedical applications by balancing structural integrity, cell compatibility and controlled immune response.

## Graphical abstract

**Graphical Abstract:**
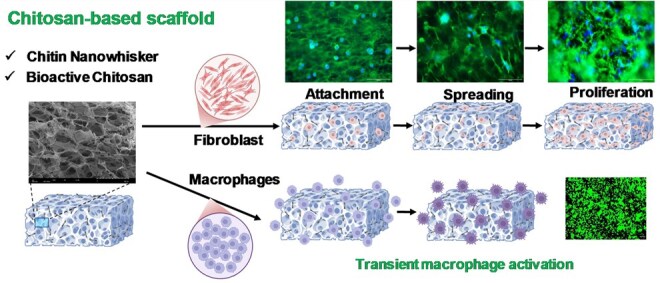

## Introduction

In recent years, interest in developing effective strategies to address tissue deterioration caused by aging, injuries or tumors has increased [1]. Today, one of the main approaches to treating these conditions involves autografts or allografts, *viz.* tissue taken from the patient body, or from another person, a donor, respectively. However, these procedures are complex and often require long waiting periods [2]. This has led to the need for alternative strategies that can surpass conventional methods.

Tissue engineering plays a fundamental role in the regeneration of tissues such as cartilage, bone and skin. It aims to develop scaffolds that not only support tissue regeneration but also promote cellular interaction, facilitating proliferation and differentiation [3]. These scaffolds must be biocompatible, present controlled porosity to allow the diffusion of nutrients and gases and be biodegradable to gradually integrate with the regenerated tissue without causing adverse effects. Furthermore, the ability to release bioactive substances, such as growth factors, in a controlled manner is a key benefit for many application scenarios. Various materials have been evaluated for scaffold fabrication, both synthetic and natural. Natural polymers have emerged as promising materials in tissue engineering as are produced by living organisms [4–6] Their intrinsic biodegradability, biocompatibility and the similar structural components of the extracellular matrix make them ideal environment for supporting cell adhesion and proliferation, without significant risk of cytotoxicity or adverse immune responses [7].

Of the many natural polymers studied for these uses, chitosan (CS) is one of the most abundant biomaterials in nature, which has gained considerable attention due to its exceptional properties. This polysaccharide, derived from chitin, is biocompatible, biodegradable and nontoxic [8]. CS has been investigated as a biomaterial due to its ability to form hydrogels, membranes and porous structures that provide a favorable environment for cell growth [9]. Additionally, its cationic character enables interaction with other polar molecules, along with its solubility in acidic media, allows processing under mild conditions, which facilitates scaffold fabrication with various morphologies, enhancing its biological performance in regenerative environments. CS also presents a high density of free amino groups, making it an excellent candidate for chemical modifications. This versatility allows for the incorporation of additional properties, such as antioxidant and antimicrobial activities [9], which are essential for various biomedical applications. To provide these properties to CS, an effective strategy is the modification of its amino groups with bioactive molecules. In this study, 1-methylimidazole (MeIm) was used, a compound that incorporates an imidazole ring which, when quaternized, has shown remarkable antioxidant and antimicrobial properties [10]. The imidazole ring is known for its ability to interact with reactive oxygen species (ROS) [11], making it an efficient agent to neutralize the effects of oxidative stress [12]. Moreover, quaternized versions of this ring exhibit potent antimicrobial activity, particularly against common pathogens, improving the biomedical applications of modified materials. Thus, CS modified with MeIm not only could enhance its biocompatibility but also provides antioxidant and antimicrobial properties, crucial for its use in scaffold fabrication for tissue engineering. In spite of such excellent properties, CS scaffolds present as main limitations their low mechanical strength, which limits their use in applications requiring strong structural support, such as bone or cartilage tissue regeneration [9, 13]. Insufficient strength in these materials can cause structural failure under mechanical stress, leading to premature collapse and poor integration with the host tissue [14]. Moreover, there are other factors that affect the mechanical properties, such as porosity or the scaffold’s interconnectivity, which can modify its stiffness [15].

To overcome these limitations, a promising option is the incorporation of some nanomaterials [16–18]. One of this reinforcement could be chitin nanowhiskers (Nws), which derived from the acylation of chitin and have different advantages like a high crystallinity and surface area that not only improve the scaffold’s stiffness and strength, but also can promote cell spreading, facilitating tissue regeneration [19, 20]. Furthermore, the surface of these nanowhiskers contains active functional groups, such as hydroxyl, amino and acetamido, which, combined with their rod-like structure, can interact with microbial membranes, leading to a knife effect that produces antimicrobial activity [21]. However, it is important to study this effect on cells, because it presents challenges regarding cellular biocompatibility [22]. Specifically, the penetration of these structures into cellular membranes may disrupt cellular integrity, as observed with bacterial membranes, which have both positive implications, such as antimicrobial properties, and negative effects on cellular welfare. However, even with the incorporation of nanowhiskers to improve mechanical strength and the biocompatibility of the CS, the implantation of these scaffolds into the human body can still present some difficulties. A significant issue is the potential for an immune response [23]. Following insertion, the body may recognize the biomaterial as a foreign entity, causing an inflammatory reaction. This immunological reaction can sometimes be negative, leading to chronic inflammation, poor integration with adjacent tissues or even rejection of the implant [24, 25]. Macrophages, as essential components of the immune system, have a critical role in regulating these responses. Their behavior, including the release of pro-inflammatory cytokines such as interleukin-6 (IL-6), significantly impact the healing process [26, 27]. IL-6, a cytokine involved in inflammation and immune regulation, promotes or inhibits tissue healing, depending on its levels. An excessive inflammatory response, driven by cytokine release, could delay the tissue regeneration and scaffold integration. Therefore, understanding and controlling the immune response to implanted scaffolds is essential to ensure their safety and effectiveness in tissue engineering.

In this work, a 1-methylimidazole modified CS was used to prepare CS-based scaffolds reinforced with 1 wt% chitin nanowhiskers. Its mechanical, biological and cellular properties were evaluated. Besides, the proliferation of human fibroblasts on these scaffolds was studied, assessing their biocompatibility, biodegradability and the influence of the antioxidant and antimicrobial properties of the material on cellular interaction. Additionally, the response of macrophages to the presence of these scaffolds was also investigated, focusing on the modulation of immune responses and inflammatory markers such as IL-6. This integrated approach will validate the potential of the modified scaffolds as effective platforms for tissue engineering, addressing both structural properties and biological effects, contributing to the advancement of CS’s biomedical applications.

## Materials and methods

CS derived from shrimp shells with a deacetylation degree above 75% was obtained from Merck. CS has an average molecular weight of 357 ± 10 kDa and a degree of deacetylation of 77% [28]. Chitin nanowhiskers were synthesized from the same source following previously reported methods [29]. Glycerol (99%), 6-hydroxy-2,5,7,8-tetramethylchroman-2-carboxylic acid (Trolox, 97%) and 2,2-diphenyl-1-picrylhydrazyl (DPPH) were also purchased from Merck. Genipin (Gp) and lysozyme (lyz, form egg white) were obtained from Fisher. CS modified with 1-methylimidazole was synthesized and characterized according to previously established work presents a modification degree of 50% [28]. All organic solvents used in this study were of analytical grade. Glacial acetic acid, hydrochloric acid (HCl, 37%) and methanol (MeOH) were supplied by Scharlau.

For biological evaluations, sodium chloride solution (NaCl, cell culture grade, BioXtra) and phosphate-buffered saline (PBS, pH 7.4, Merck) were used. Mueller–Hinton broth for bacterial growth was obtained from Becton, Dickinson and Company, while 96-well microplates were acquired from ThermoFisher Scientific. Columbia agar plates enriched with 5% sheep blood were sourced from Fisher. The bacterial strains used in this study included: *Escherichia coli* (*E. coli*, ATCC 25922), *Staphylococcus aureus* (*S. aureus*, ATCC 29213), *Enterococcus faecalis* (*E. faecalis* ATCC 29212) and *Pseudomonas aeruginosa* (*P. aeruginosa*, ATCC 27853) all obtained from Oxoid™.

For cell culture, Dulbecco’s phosphate-buffered saline (DPBS), Dulbecco’s modified eagle medium (DMEM) and penicillin/streptomycin (P/S) were obtained from Gibco. 3-(4,5-Dimethylthiazol-2-yl)-2,5-diphenyl tetrazolium bromide (MTT) reagent was supplied by Sigma-Aldrich. Fetal bovine serum (FBS) was purchased from PAN Biotech and it was heat-inactivated at 65°C for 30 min, while the AlamarBlue Cell Viability Reagent was sourced from Invitrogen. Sodium dodecyl sulfate (SDS, ≥99.5%), bovine serum albumin (BSA), 4′,6-diamidino-2-phenylindole, dihydrochloride (DAPI), CoraLite-488 Phalloidin (in sterile water, Proteintech), the micro bicinchoninic acid (BCA) Protein Assay Kit and the LIVE/DEAD™ Cytotoxicity/Viability Kit for mammalian cells were obtained from Thermo Fisher. Additionally, the Mouse IL-6 DuoSet Enzyme-linked Immunosorbent Assay (ELISA) Kit was from R&D Systems. Paraformaldehyde (PFA, 4%) was obtained from Thermo Fisher, QuantiBlue reagent was purchased from InvivoGen and the LiVE/DEAD staining kit was obtained from Thermo Fisher Scientific. The cell culture used in this study included: human dermal fibroblast adult (NHDF-Ad) and murine macrophages (J774 Dual cells) obtained from InvivoGen.

### CS scaffold preparation

**Figure 1:**
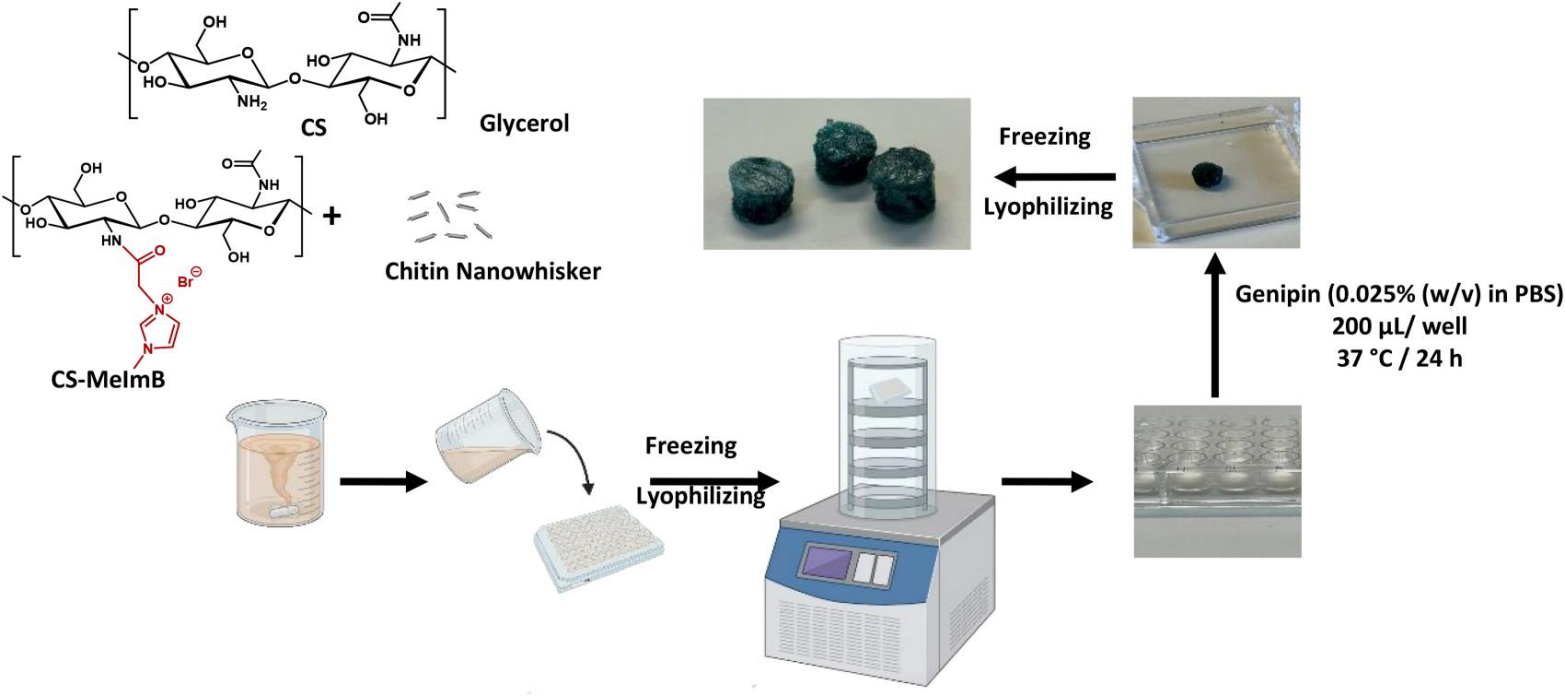
Schematic representation of scaffold preparation.

Scaffolds were prepared using a 1% (w/v) polymer solution in acetic acid (1% v/v). The formulation consisted of 85% polymer and 15% glycerol as plasticizer, with the polymer fraction composed of CS, 10% CS modified with MeImB (CS-MeImB), and 1% chitin nanowhiskers (Table 1). The respective amounts of each component were dispersed in the acetic acid solution and stirred continuously at room temperature for 24 h to ensure homogeneous distribution (Figure 1). After this time, the resulting mixture was transferred into a 96-well plate, which was frozen overnight at −20°C in a freezer. Also, glycerol was added to control the ice crystal formation during the freezing, reducing the development of sharp crystals that could damage the porous structure [30]. This contributes to the formation of more rounded and uniform pores. Subsequently, the samples were lyophilized in 96-well plates under controlled conditions.

**Table 1:** Composition of chitosan scaffolds (%).

Sample	CS (%)	CS-MeImB (%)	Nw (%)	Gly (%)
s-CS	85	–	0	15
s-CS-Nw	84	–	1	15
s-CS-MeImB	75	10	0	15
s-CS-MeImB-Nw	74	10	1	15

Following lyophilization, the scaffolds were crosslinked with genipin at a concentration of 0.025% (w/v) in PBS [31]. The scaffolds were immersed in the genipin solution and incubated at 37°C for 24 h. After the crosslinking process, the scaffolds were washed with PBS twice and subsequently transferred to ethanol for 12 h. This crosslinking step resulted in a visible color change from white to dark blue and a final dimension of 5 mm diameter and 2 mm thickness.

### Microstructural characterization

The microstructural features of the scaffolds were analyzed using scanning electron microscopy (SEM). A Hitachi S-8000 in transmitted electron imaging mode was employed to determine pore size and morphology. Both surface and internal porosity were examined. For internal structure visualization, the scaffolds were fractured after immersion in liquid nitrogen. Prior to imaging, the samples were sputter-coated with Au/Pd (80/20) layer to enhance conductivity. The pore size of scaffolds was measured by using an image analysis software (ImageJ) of SEM micrographs. Manual mode of the ImageJ analyzers in the vertical of each pore was used for estimation of the average diameter at least in 150 pores of five SEM micrographs for each scaffold type.

### Porous microcarriers

The interconnectivity of the scaffold porosity was assessed using the liquid displacement method. Three replicates of each scaffold type, with an initial weight (*W*_i_), were immersed in 0.5 mL of ethanol (density 0.789 g/cm^3^ at 20°C) for 30 min. Following incubation, the scaffolds were removed, excess liquid was drained and they were weighed again to obtain the final mass (*W*_f_) [32–34]. The degree of porosity interconnectivity was then calculated using Equation (1)


(1) $$\mathrm{Interconectivity}\;(\%)\;=\;(\frac{W_{f}-W_{i}}{\rho_{\mathrm{EtOH}}\times V_{i}})\times 100$$

### Swelling capacity

The swelling capacity of the scaffolds was evaluated in DMEM. Three replicates of each scaffold type were independently immersed in the culture medium and incubated at 37°C [35]. After 2.5 h, the scaffolds were weighed to determine their swelling using Equation (2)


(2) $$\mathrm{Swelling}\;(\%)\;=\;(\frac{W_{f}-W_{i}}{W_{i}})\times 100$$

### Antioxidant properties

The antioxidant activity of the CS scaffolds was evaluated using the *in vitro* colorimetric system with the free radical DPPH [36]. To prepare the DPPH solution, 15 mg of DPPH was dissolved in 250 mL of MeOH. Then, 1 mL of this DPPH solution was added to each vial containing one of scaffold. All experiments were conducted in the absence of light to prevent any photochemical reactions that could interfere with the measurement.

The absorbance of the DPPH solution was measured at *λ* = 517 nm, the maximum absorption wavelength for DPPH. Absorbance readings were taken hourly during the first 8 h, followed by additional measurements at 16 and 24 h. Throughout the experiment, the scaffolds were kept in darkness. The antioxidant capacity of the scaffolds was determined by calculating the percentage of radical inhibition, as described in Equation (3)


(3) $$\mathrm{Inhibition}\;(\%)\;=\;(\frac{{\mathrm{Absorbance}}_{\mathrm{control}}-{\mathrm{Absorbance}}_{\mathrm{sample}}}{{\mathrm{Absorbance}}_{\mathrm{control}}})\times 100$$

### Antimicrobial properties

The antimicrobial properties of s-CS and s-CS-MeImB, both with and without nanowhiskers, were evaluated following a methodology based on the E2149-20 standard method from the American Society for Testing and Materials (ASTM) [37]. The assays were conducted against *S. aureus, E. faecalis, E. coli* and *P. aeruginosa* strains.

For each sample, one scaffold was immersed in 9 mL of PBS. Subsequently, 1 mL of a microbial suspension at a concentration of ca. 10^6^ CFU/mL was added to the solution containing the scaffold. The tubes were incubated under agitation at 100 rpm for 24 h at 37°C.

After incubation, samples were taken to assess microbial viability. The resulting solutions were plated onto Columbia agar supplemented with 5% sheep blood and incubated at 37°C for 24 h. The antimicrobial activity was determined by counting method calculating the percentage reduction in viable microorganisms compared to the control. All measurements were performed at least in triplicate.

### Rheological properties

Mechanical properties of the scaffolds were evaluated through rheological characterization after hydration in PBS for 24 h at room temperature. Measurements were performed using an MCR702e MultiDrive rheometer (Anton-Paar, Austria) equipped with a stainless-steel 8 mm parallel plate geometry that fit the size of the scaffolds. The gap height was adjusted to ensure proper scaffold contact while avoiding excessive compression (0.5 N maximum normal force).

Oscillatory amplitude sweeps were performed in a strain range of 0.1–100% at a constant angular frequency of 10 rad/s. The storage modulus (*G′*) and loss modulus (*G*″) were recorded. Each condition was measured with 10 replicates and the average ± standard deviation is reported.

### Protein adsorption

The protein adsorption capacity of the scaffolds was evaluated using the MicroBCA™ Protein Assay Kit, following the manufacturer’s instructions. A calibration curve was prepared using BSA standards at different concentrations (0–200 µg/mL) in PBS. Each scaffold was placed in a well of a 96-well plate and incubated with 100 µL of a 200 µg/mL BSA solution in PBS at 37°C for 2 h to allow protein adsorption. After incubation, scaffolds were carefully washed twice with PBS to remove reversibly adsorbed protein and transferred to new wells to eliminate any residual protein solution adhered to the plate walls. For the protein quantification, 200 µL of 1% SDS in PBS was added and incubated for 30 min at 37°C under gentle agitation to disrupt protein–surface interactions and ensure complete desorption [38]. Following desorption, 150 µL of the SDS–protein solution was mixed with 150 µL of the MicroBCA working reagent in a 96-well plate and incubated at 37°C for 2 h in the dark. Absorbance was measured at 562 nm using a microplate reader (TECAN Spark). The protein adsorption was calculated by comparing the protein concentration in the desorbed solution with the initial BSA concentration, using the calibration curve for quantification. All experiments were conducted in triplicate, and results were expressed as the amount of protein adsorbed per scaffold surface area.

### Cytotoxicity and proliferation studies

The cytotoxicity and proliferation of normal human adult dermal fibroblasts (NHDF-Ad, purchased from Lonza, Germany) on the CS-based scaffolds were evaluated. These cells were cultured in growth media (FBM, Fibroblast Growth Basal Media, Lonza, Germany) and supplemented with 1% P/S and FGM-2 SingleQuots supplements (containing insulin, hFGF-B, GA-1000). All cells were incubated at 37°C and 5% CO_2_.

The scaffolds were sterilized under UV light for 30 min on each side and then, hydrated with FBS for 30 min at room temperature to enhance fibroblast adhesion within their porous structure. A total of 10 000 fibroblasts were seeded onto each scaffold, which were placed in individual wells of a non-treated 96-well plate. Two control groups were included: one without cells (negative control) and another with fibroblasts cultured in a treated 96-well plate to ensure proper cell adhesion. After 24 h of incubation, the scaffolds with fibroblasts were transferred to a new 96-well plate to ensure that only cells adhered to the scaffolds were analyzed, eliminating any cells that might have adhered to the well walls.

Cell viability was assessed using the Alamar Blue assay at several time points: 1, 3, 7, 14 and 21 days. For the Alamar Blue assay, 10% (v/v) of the Alamar Blue reagent was added directly to each well containing the scaffolds and cells [39]. After adding the reagent, the plate was incubated at 37°C in a humidified incubator for 2 h.

After the incubation period, the media was then transferred to black 96-well plates. Fluorescence (Ex/Em 570/600 nm) was measured using a plate reader. Controls of cells and blanks with only media were also analyzed. The results were analyzed to observe changes in cellular activity and proliferation across the time points.

### Cell staining

To investigate fibroblast adhesion and proliferation on the scaffolds, cell cytoskeleton and nucleus were stained at specific time periods (1, 2, 3, 7, 14 and 21 days). First, the culture medium was discarded, and the scaffolds were fixed with 200 µL of 4% PFA to each well, ensuring complete scaffold coverage. The 96-well plate was incubated for 1 h at 4°C to preserve the cellular structure of the fibroblasts adhered to each scaffold. PFA cross-links proteins, stabilizing the cytoskeleton and preventing cellular degradation during subsequent staining steps. After fixation, scaffolds were washed three times with PBS to remove residual fixative.

To minimize nonspecific binding of fluorescent dyes, a blocking step was performed by incubating the scaffolds with a 1% BSA solution in PBS. This step helps to prevent background fluorescence by occupying nonspecific binding sites on both the scaffold surface and cellular structures. For cytoskeletal and nuclear staining, a staining solution was prepared in PBS containing Phalloidin-iFluor 488 at a 1:1000 dilution and DAPI at a 1:1000 dilution. Phalloidin-iFluor 488 selectively binds to filamentous actin (F-actin), allowing visualization of the cytoskeleton, while DAPI intercalates with DNA, staining cell nuclei. The staining solution was added to each well, ensuring complete coverage of the scaffold, and incubated at room temperature for 1 h in the dark to prevent photobleaching. Finally, the scaffolds were washed three times with PBS before fluorescence imaging. This staining protocol allowed for the assessment of fibroblast distribution, morphology and proliferation on the scaffolds over time. This was performed using the Leica DMI6000 B microscope at a 20× of magnification.

### Fibroblast morphology analysis

The morphology of fibroblasts attached to the scaffolds was examined using SEM imaging. After fixation with PFA, the scaffolds remained immersed in PBS following previous treatments. To ensure proper imaging, a dehydration process was performed using a graded ethanol–PBS series, ending with 100% ethanol [40]. The samples were then air-dried for 24 h. Once completely dry, they were sputter-coated with an Au/Pd (80/20) layer and imaged using the same SEM system as before.

### Macrophage response to scaffolds

The immune response to the scaffolds was assessed using macrophages. The murine cell line J774-Dual was used as *in vitro* model. Cells were cultured in DMEM supplemented with 10% FBS and 1% P/S. The cultures were maintained at 37°C in a humidified 5% CO_2_ atmosphere.

Cells were seeded at a density of 10 000 in 100 µL per well in a treated 96-well plate and incubated for 24 h at 37°C. After incubation of the cells for 24 h, the culture medium was removed, and 100 µL of fresh growth medium was added to each well, followed by the addition of the UV-sterilized scaffolds.

For comparative analysis, different experimental groups were included. Scaffolds with growth medium but without cells served as a negative control, while macrophages stimulated with LPS at 15 ng/mL were used as a positive control. The cells in contact with the scaffold and the controls were incubated for additional 24 h (37°C, 5% CO_2_). Following incubation, 150 µL of culture supernatant was collected from each well to quantify IL-6 cytokine secretion via ELISA. J774-Dual cells are genetically modified to express a secreted embryonic alkaline phosphatase (SEAP) under the NFkB promoter, which can be detected colorimetrically using the QuantiBlue assay.

### QuantiBlue assay

The early immune response of J774-dual macrophages in contact with scaffolds was evaluated by quantifying SEAP levels using the QuantiBlue assay. SEAP is a stable secreted enzyme whose production is triggered by the activation of the NFκB pathway, helping to evaluate macrophage activation. Once the QuantiBlue reagent was prepared according to the manufacturer’s instructions, 180 μL of the solution was dispensed into a fresh 96-well plate. Then, 20 μL of the previously collected culture supernatant was added to each well. The plate was incubated at 37°C for 30 min, protected from light. Absorbance was then measured at 620–655 nm using a microplate reader [41]. Two control groups were also included in the experiment, consisting of macrophages cultured in standard medium without scaffolds as a negative control, and macrophages stimulated with LPS, 0.1 μg/mL, to serve as a positive control for activation.

### ELISA for IL-6 quantification

The quantification of IL-6 secretion was performed using a Mouse IL-6 DuoSet ELISA Kit, following the manufacturer’s instructions [42]. Briefly, a 96-well microplate was coated with 50 µL of capture antibody per well and incubated overnight at room temperature in the dark. The next day, the plate was washed three times with wash buffer to remove unbound antibody. After the final wash, any remaining buffer was completely removed, and 300 µL of reagent diluent was added to each well for blocking nonspecific binding sites. The plate was incubated at room temperature for 1 h, followed by three additional washes with wash buffer. After blocking, 100 µL of the collected supernatants and the standards were added to the respective wells and incubated for 2 h at room temperature. Following incubation, the washing step was repeated, and 50 µL of detection antibody was added to each well. The plate was incubated for other 2 h at room temperature before washing three times with wash buffer. Next, 100 µL of streptavidin–HRP was added to each well and incubated for 20 min at room temperature. The plate was then washed again before adding 100 µL of substrate solution to each well. The reaction was allowed to develop for 20 min, protected from light at room temperature. Finally, 50 µL of stop solution was added to each well, and the absorbance was measured at 450 nm, with a 540 nm wavelength correction, using a microplate reader. All samples, standards and controls were analyzed in triplicate, and cytokine concentrations were determined using a standard curve generated from recombinant murine IL-6 expressed in pg/mL.

### Live/dead viability assay

Macrophage viability after 24 h of scaffold exposure was evaluated using the LIVE/DEAD™ Viability/Cytotoxicity Kit, which differentiates viable from non-viable cells based on fluorescence emission [43]. The culture medium was carefully removed from each well and washed twice with PBS. To assess viability, cells were stained with fluorescein diacetate (FDA), 5 mg/mL for live cells and propidium iodide (PI), 2 mg/mL, for dead cells. The final concentrations of FDA and PI in PBS were adjusted to 40 and 30 μg/mL, respectively. A total of 100 μL of the prepared staining solution was added directly to the cells and the plate was incubated for 15 min at 37°C, protected from light. After incubation, fluorescence images were acquired using a Keyence BZ-X810 All-in-one fluorescence microscope, capturing a minimum of 10 images per sample in multiple fields to ensure representative analysis. The viability was determined by counting live (green) and total (green + red) cells in the images. The percentage of live cells was calculated as follows in Equation (4):


(4) $$\mathrm{Viability}\;(\%)\;=\;\frac{\mathrm{no}\;\mathrm{live}\;\mathrm{cells}}{\mathrm{total}\;\mathrm{cells}}\times 100$$

### *In vitro* biodegradation studies

The enzymatic biodegradation assay was performed using the previously prepared scaffolds, which were sterilized by UV light for 30 min on each side. Lysozyme was used at a concentration of 13 mg/L in PBS, corresponding to the approximate concentration found in the human body [44, 45]. Three replicates of the different scaffolds, with an initial weight (*W*_0_), were immersed in the lysozyme solution at 37°C with 60 rpm of agitation. Measurements were first taken at 3 days and then, weekly until 63 days (9 weeks), with the lysozyme medium being changed every 72 h. After the designated periods, the scaffolds were removed from the medium, lyophilized and weighed to determine their final weight (*W*_1_). Scaffold degradation was calculated using Equation (5)


(5) $$\mathrm{Weight}\;\mathrm{lost}\;(\%)\;=\;(\frac{W_{0}-W_{1}}{W_{0}})\;\times \;100$$

To evaluate the potential cytotoxicity of the degradation products released during the biodegradation, a complementary viability assay was performed using NHDF-Ad. Specifically, the media were collected from the enzymatic degradation assay at 7, 28 and 63 days, representing the first, the middle and the last scaffold decomposition and stored at 4°C until use. These media contained possible soluble degradation products resulting from lysozyme activity on the scaffold structure.

NHDFs were seeded at a density of 20 000 cells per well in a 96-well plate treated and then, the plate was incubated for 24 h at 37°C with 5% CO_2_ to allow for cell attachment and growth [46]. After incubation, the culture medium was removed and replaced with 100 µL of fresh medium and 50 µL of the degradation medium collected. Cells were incubated with these conditioned media for an additional 48 h; cells incubated without degradation media were used as a blank and the negative control were cells incubated with the lysozyme medium without scaffold. All the samples were seeded in triplicate for each condition. Following exposure, cell viability was evaluated using the MTT assay. After 24 h, 15 µL of MTT solution (10% of total volume) was added to each well and incubated for 2 h at 37 °C. Subsequently, the medium was carefully removed, and 100 µL of DMSO was added to solubilize the formazan crystals formed by metabolically active cells. Absorbance was measured at 570 nm using a microplate reader. Cell viability was calculated relative to a control group of the cells exposed only to fresh medium, allowing for the assessment of any cytotoxic effects potentially caused by the degradation products of the scaffolds.

### Statistical analysis

To determine whether there were significant differences between the groups in the interconnectivity, swelling capacity and cytotoxicity assays, a one-way analysis of variance (ANOVA) was conducted. Subsequently, Tukey’s test was applied. A significance level of *P* ≤ 0.05 was considered. For the rest of measurements, arithmetic mean and standard deviation was performed. In all the cases a minimum of triplicate samples were analyzed.

## Results and discussion

### Material physicochemical characterization

**Figure 2:**
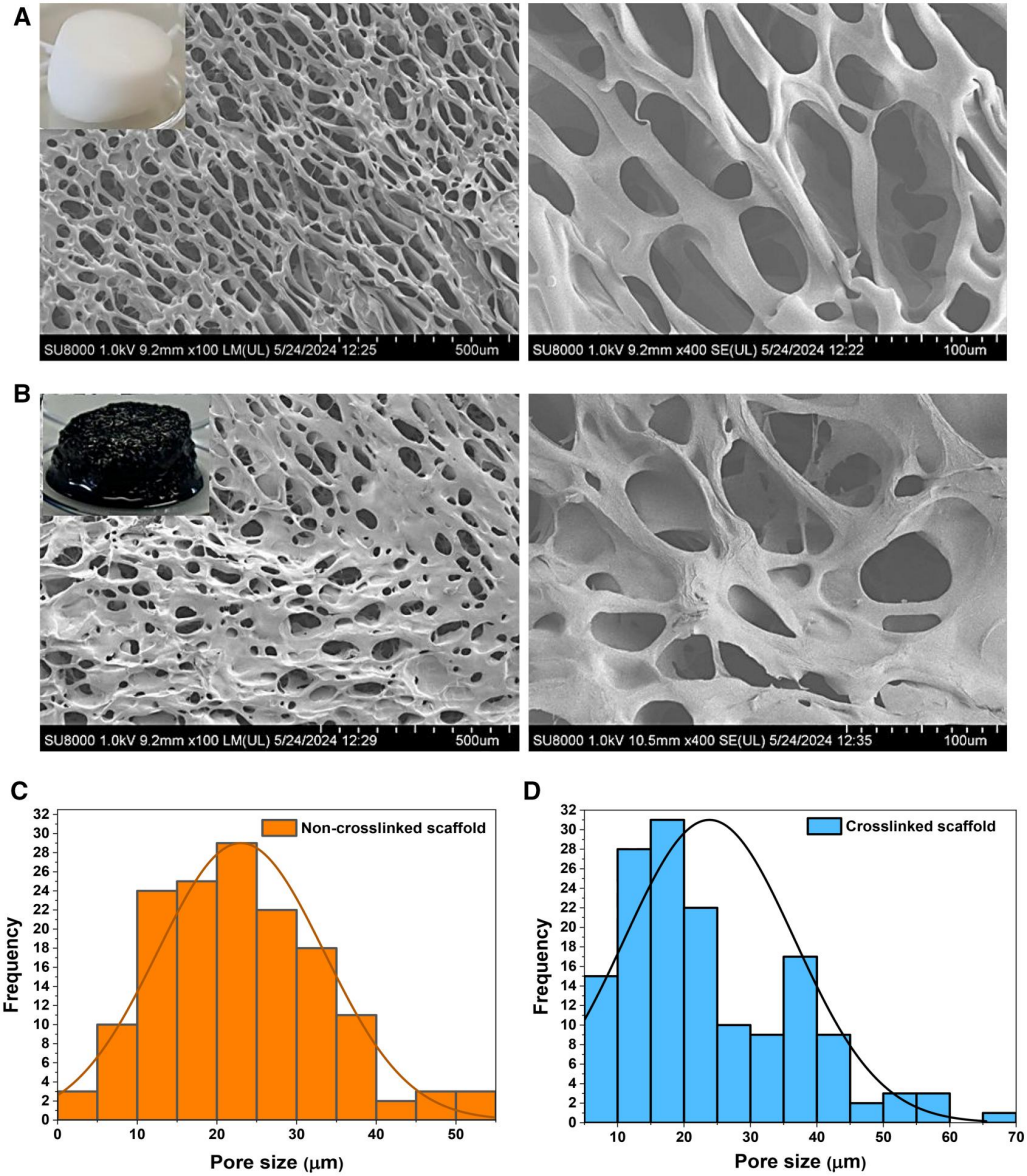
Microstructural characterization. Scanning electron microscopy images of the non-crosslinked (**A**) and genipin-crosslinked (**B**) scaffolds (scale bars 500 µm (left), 100 µm (right) respectively). Top left insets depict the scaffolds macroscopically. (**C**, **D**) Frequency distribution of pore length measurements for non-crosslinked (**C**) and crosslinked (**D**) scaffolds (*n* = 150).

First, CS scaffolds (non-crosslinked and genipin-crosslinked) were fabricated as porous sponges after lyophilization, and the microstructure obtained was characterized (Figure 2). Differences in microstructure between non-crosslinked and genipin-crosslinked scaffolds were observed (Figure 2A and B). Pore shape of non-crosslinked scaffolds appeared more elongated compared to the genipin-crosslinked ones, which presented a rounder shape. Pore density was also different, obtaining a higher number of pores in the non-crosslinked scaffold. This could be attributed to the effect of genipin crosslinking of the CS chains [47].

The quantification of the pore size (Figure 2C and D) revealed that the range of pore width for non-crosslinked scaffolds was between 10 and 40 μm, with the most frequent size (27% of the pores) falling between 20 and 25 μm. In comparison, the pore width range for the crosslinked scaffold spans in the same range; however, the distribution of pore sizes was less homogeneous. The most frequent size (30% of the pores) between 15 and 20 μm. Pore distribution in the non-crosslinked scaffold was more uniform compared to the crosslinked scaffold. In literature, pores smaller than 50 μm are shown to improve the mechanical properties of scaffolds; however, smaller pores tend to lower cellular viability due to restricted diffusion of nutrients and cell colonization and proliferation could be hindered [48]. In addition, for improved integration of the scaffold *in vivo*, larger pores are more compatible with capillary penetration [49]. In this case, the scaffolds predominantly have moderate pores (15–20 μm) allowing cell migration, nutrient transport and deposition of ECM, whereas some bigger pores present in the structures could facilitate blood vessels [49]. Once the impact of genipin crosslinking on the microstructure of the CS scaffolds was studied, the porosity of the other scaffold formulations was also evaluated (Table 2).

**Table 2:** Evaluation of pore diameter of all CS-based crosslinked scaffolds.

Sample	Average pore diameter (μm)	Range of pore size (μm)
s-CS	16.6 ± 6.9^a^	10–30
s-CS-MeImB	22.2 ± 10.3^b^	15–25
s-CS-Nw	17.9 ± 8.3^ac^	10–25
s-CS-MeImB-Nw	21.7 ± 11.1^bc^	15–25

Values denoted with the same letter are not significantly different for the Tukey test (significance level of *P* ≤ 0.05).

The different scaffold formulations showed distinct microporosity. Chemical modification with MeImB (CS-MeImB) resulted in a slight increase in pore size compared to the unmodified CS scaffold. This effect could be attributed to the presence of the positively charged group MeImB, which could interfere with the polymer structure and the uniform nucleation of ice crystals during freeze-drying, resulting in a less compact system and slightly larger pores [50]. In contrast, the incorporation of nanowhiskers did not significantly modify the pore size distribution [51], either combined with s-CS nor s-CS-MeImB.

**Figure 3:**
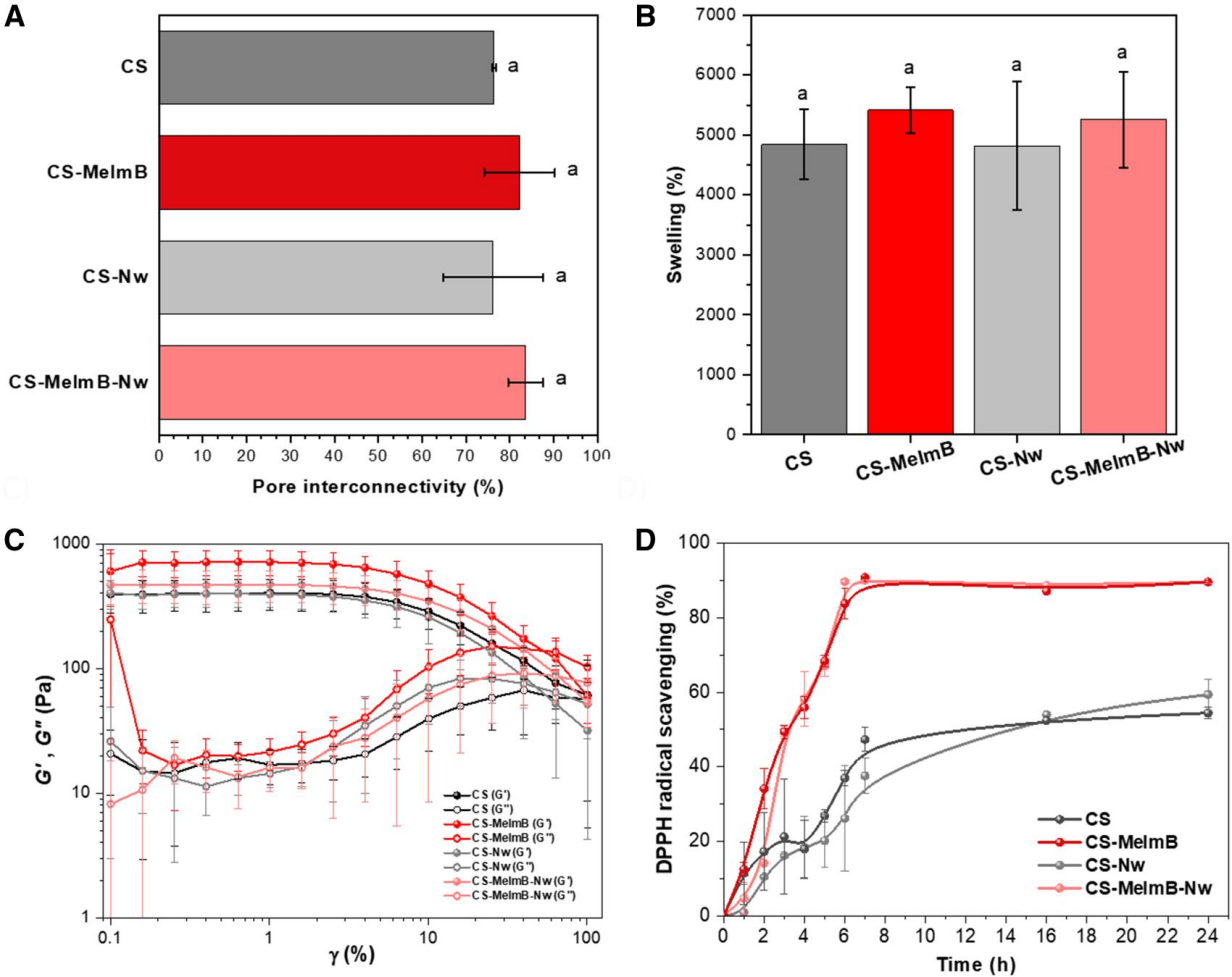
Scaffold properties. (**A**) Percentage of pore interconnectivity; (**B**) cell culture medium uptake (%); (**C**) amplitude sweep of scaffolds and (**D**) antioxidant activity of the scaffolds. For all conditions: s-CS (dark grey), s-CS-MeImB (red), s-CS-Nw (light grey) and s-CS-MeImB-Nw (light red).

The interconnectivity of the pores was also studied. Higher interconnectivity enables better diffusion of nutrients, growth factors and oxygen, as well as the removal of waste products [52]. Liquid displacement was used to study the interconnectivity and results are shown in Figure 3A. All scaffolds exhibited a high percentage of interconnectivity, between 70% and 80%, which is within the range of interconnectivity that has been shown to promote the survival and proliferation of cells in 3D scaffolds. Some studies show that high scaffold interconnectivity combined with a small pore size allow cell adhesion and proliferation [33]. Slight variations were observed among conditions; however, these were not statistically significant.

Another essential parameter is high-water swelling capacity. This is necessary to create a favorable environment for the cells, where the water and oxygen diffusion can easily allow the exchange of nutrients and waste through the scaffold. Swelling can be influenced by factors such as the degree of crosslinking, polymer content or pore size, among others [53]. The swelling capacity of the scaffolds was measured in cell culture medium (DMEM), which mimics physiological-like conditions such as osmolarity or pH and it is more representative *in vivo* environments. Scaffolds were immersed in DMEM for two and a half hours (Figure 3B), since it was determined that this is the time necessary to reach the equilibrium. The scaffolds modified with MeImB presented a slightly higher swelling capacity. This could be due to its high hydrophilic character, its slightly higher average pore diameter and its higher pore interconnectivity; however, the differences were not statistically different from the other scaffolds tested. The intrinsic high-water uptake capacity of polysaccharides such as CS governs water uptake, and the differences in CS content between conditions are not high. This is in alignment with other studies [54].

Mechanical properties of the scaffolds were studied by means of oscillatory rheology (see amplitude sweep curves for the studied samples in Figure 3C). At low strains, linear viscoelastic (LVE) region, all scaffolds presented a gel-like behavior (storage modulus, *G*′, larger than the loss modulus, *G*″), up to ∼4% strain, where the transition from linear to non-linear behavior occurred (yielding). The crossover of *G*′ and *G*″ occurred at strains >10%, significantly larger than that for rigid gels, where the crossover occurs at strains <0.1% [55]. The studied scaffolds presented slight differences in the *G*′ values in the LVE region. s-CS-MeImB presented an average *G*′ around 700 ± 160 Pa, higher than s-CS (398 ± 109 Pa), s-CS-Nw (395 ± 87 Pa) and s-CS-MeImB-Nw (470 ± 136 Pa). Interestingly, the reinforcement effect from chitin nanowhiskers was negligible, contrary to our original hypothesis [20, 56]. This could be due to the relatively low wt.% of nanowhiskers used. Using *G*′ values at the LVE region, the Young’s modulus (*E*) for each scaffold assuming a Poisson’s ratio (*ν*) of 0.5 was calculated according to:


(6) E=2G′(1+ν)

In all cases, the *E* < 2.1 ± 0.5 kPa, revealing that the studied scaffolds are sufficiently soft to growth soft tissues. The tan*δ* calculated within the LVE regime showed that statistically s-CS scaffolds (0.0426 ± 0.0041) present a slightly more viscous behavior compared to the modified scaffolds s-CS-Nw (0.0290 ± 0.0039), s-CS-MeImB (0.0347 ± 0.0049) and s-CS-MeImB-Nw (0.0344 ± 0.0043).

### Antioxidant properties

The antioxidant properties were also evaluated via DPPH radical scavenging activity quantification (Figure 3D) [57]. Strong antioxidant activities are desirable in scaffolds for tissue engineering applications as ROS are generated at the implantation site and their neutralization reduces inflammatory response and promotes cell proliferation and differentiation [58]. As expected, scaffolds containing MeImB-modified CS achieved the highest inhibition percentage (around 90%) within 6–7 h. In contrast, the scaffold made of pure CS or CS-Nw exhibited lower antioxidant capacity, requiring a longer time to reach its maximum inhibition percentage (just under 60%). Therefore, the incorporation of 10% of CS-MeImB induces a large increase in the antioxidant activity, comparable to other results found in literature, such as crosslinked CS using tannic acid [59, 60].

### Antimicrobial properties

Antimicrobial properties are a key factor in tissue engineering, as preventing bacterial infections is crucial for the success of scaffolds and implants designed for biomedical applications. In this study, the antimicrobial activity of the scaffolds was evaluated against *S. aureus*, *P. aeruginosa*, *E. coli* and *E. faecalis* bacteria.

Modified CS with 1-methylimidazole has previously demonstrated remarkable activity against Gram-positive and Gram-negative bacteria [28], likely due to the inherent positive charge of the derivative polymer, which interacts with bacterial membranes and compromises their integrity.

Results (Table 3) showed that CS and CS-Nw scaffolds exhibited some activity against *S. aureus*, although it was not sufficient to eliminate the bacteria. In contrast, s-CS-MeImB demonstrated total bactericidal activity against *S. aureus*. Studies on these bacteria have shown that antimicrobial activity also strongly depends on pH. When the pH decreases sufficiently and the amino group of CS becomes protonated (i.e. in our case), the antimicrobial properties of the scaffolds become more effective [61]. Nevertheless, it is clear that the incorporation of 1-methylimidazole improves such activity.

**Table 3:** Antimicrobial activity of scaffolds.

	*S. aureus*	*E. faecalis*	*P. aeruginosa*	*E. coli*
s-CS	75.3 ± 1.1^a^	–	–	–
s-CS-MeImB	99.9 ± 1.4^b^	99.99 ± 0.01^b^	99.999 ± 0.001^b^	99.999 ± 0.001^b^
s-CS-Nw	79.7 ± 0.9^a^	–	–	–
s-CS-MeImB-Nw	99.999 ± 0.001^b^	99.98 ± 1.1^b^	99.999 ± 0.001^b^	99.999 ± 0.001^b^

Values having the same letter are not significantly different for Tukey test (significance level of *P* ≤ 0.05).

Regarding Gram-negative bacteria, the s-CS and s-CS-Nw scaffolds showed no significant antimicrobial activity. Conversely, s-CS-MeImB eliminated all bacterial colonies, indicating a substantial improvement in antimicrobial performance compared to unmodified CS. These findings suggest that the modification of CS with MeImB significantly enhances its antibacterial properties, making it a promising candidate for scaffolds intended for tissue regeneration. The interaction between scaffolds and different bacteria strains has been extensively studied, showing that stronger electrostatic interactions between the positively charged scaffold and the negatively charged bacterial cell wall are associated to increased bactericidal activity [62, 63].

### Cell–scaffold interactions

**Figure 4:**
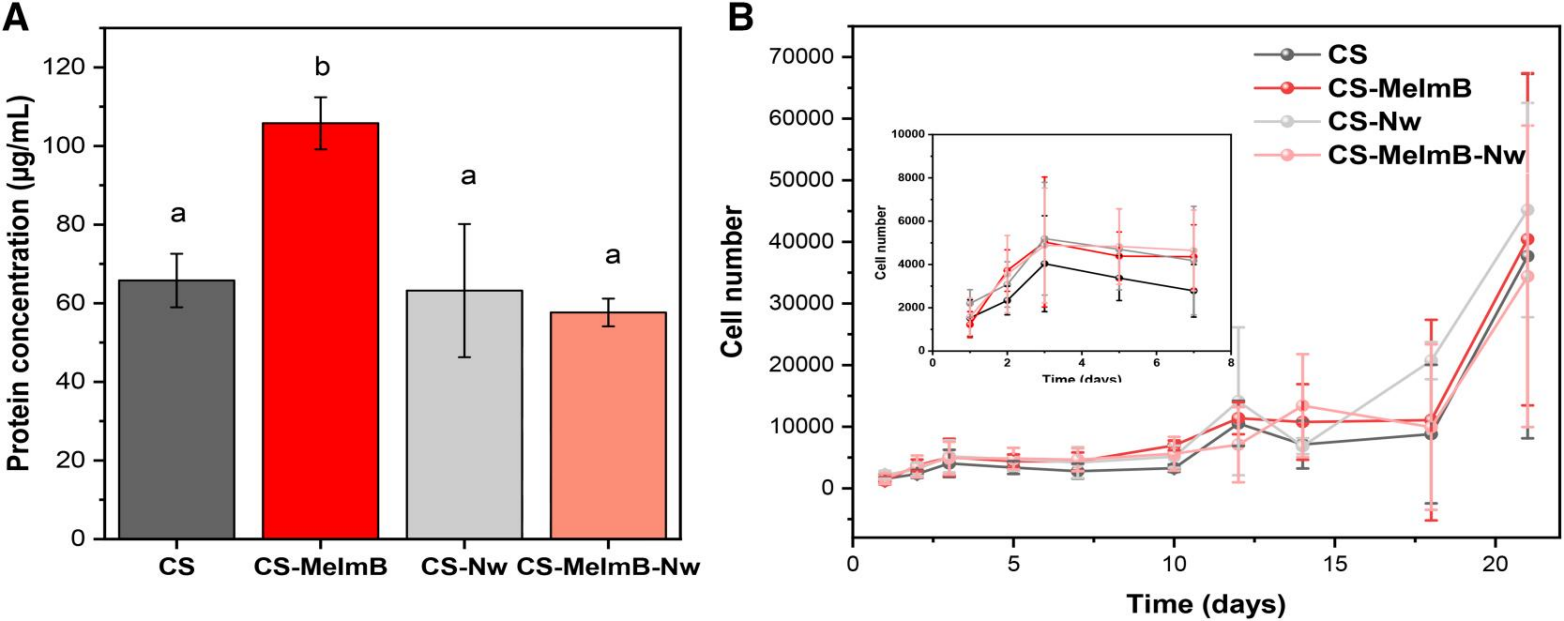
Fibroblast adhesion and proliferation. (**A**) Protein adsorption to the scaffolds. (**B**) Normal human dermal fibroblast cell proliferation over time (*n* = 3). s-CS (black), s-CS-MeImB (red), s-CS-Nw (light grey) and s-CS-MeImB-Nw (light red).

After investigating the physicochemical properties, antioxidant and antimicrobial features of the scaffolds, cell–scaffold interactions were investigated. First, protein adsorption was quantified, as it plays a crucial role in early cell attachment to biomaterials (Figure 4A). Scaffolds containing CS-MeImB exhibited a higher protein adsorption capacity compared to CS and Nw-reinforced scaffolds. This enhancement could be attributed to the additional positive charge inherent to the modified polymer, which increases the scaffold’s polarity, thereby, facilitating protein adsorption [64]. Interestingly, s-CS-MeImB-Nw did not present similar protein adsorption to s-CS-MeImB, which could indicate that the Nw might be accumulating at the CS surface, limiting protein interaction with the MeImB groups [65]. Nevertheless, this did not hinder the antioxidant activity conferred by MeImB moieties (Figure 3D). After confirming that the scaffolds allowed sufficient protein adsorption (in the μg/mL range), initial cell attachment and proliferation over longer timepoints were studied (Figure 4B).

Fibroblast cells were seeded on top of the scaffolds and their metabolic activity was tracked over time using Alamar Blue assays. First proliferation was assessed over 7 days (Figure 4B, inset), and then, the assay was extended up to 21 days to assess colonization of the scaffolds. All scaffolds presented a similar cell number at 24 h, indicating that cell viability and cell adhesion were similar among the different conditions tested. This indicates that the differences observed in protein adsorption did not affect early cell adhesion. Cells rapidly proliferated within the first 3 days after seeding, followed by a plateau phase. This stabilization in proliferation is typically seen in confluent layers of cells, where cell contact inhibition is triggered. This could be due to the cells occupying the surface of the scaffold [66].

To further investigate if cells were able to colonize the scaffold, another assay was conducted for 21 days. The results revealed growth during the first 3 days followed by a plateau from day 3 to day 10. Then, another increase in cell number was observed, which plateaued up to day 17, from which a significant rise in cell number was observed. The later increase in cell growth at days 10 and 17 could be related to cells gradually colonizing the scaffold, where migration and proliferation slow down as the cells must migrate and degrade the scaffold. Similar delayed adhesion patterns have been reported for biomaterials requiring prolonged cell interactions with the materials before optimal proliferations were achieved [67]. These findings highlight the importance of extended incubation times when evaluating scaffold biocompatibility and cellular responses in tissue engineering applications.

### Cell morphology

**Figure 5:**
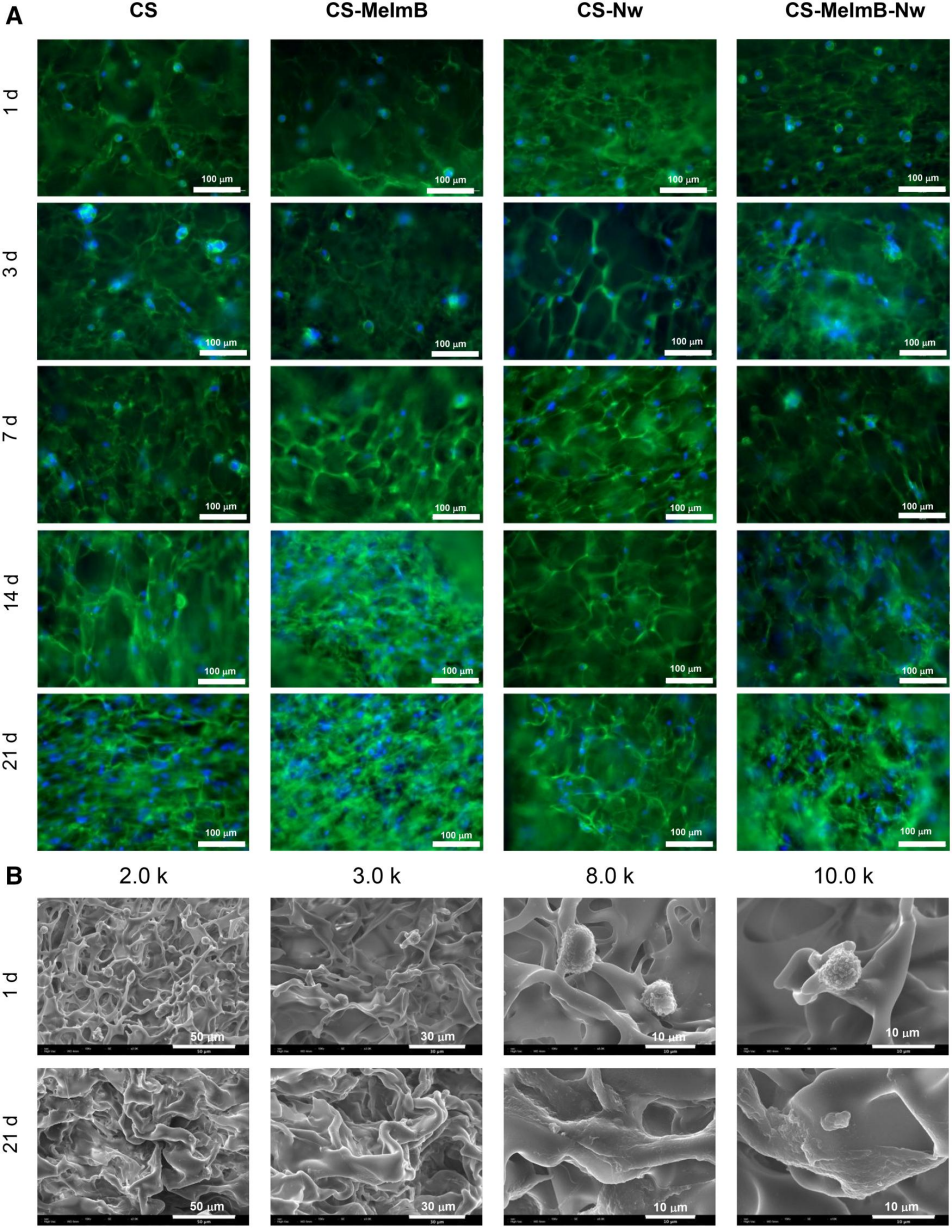
Fibroblast morphology characterization. (**A**) Panel depicts representative images of human dermal fibroblasts at different timepoints and on different scaffolds (green: actin cytoskeleton, blue: nucleus) (scale bar: 100 μm). (B) Representative SEM images of all scaffolds at days 1 and 21.

Following the evaluation of fibroblast proliferation on the scaffolds, cell morphology was investigated at different timepoints via staining of the cytoskeleton (Figure 5A) [68]. At 24 h, fibroblasts exhibited a rounded morphology with minimal attachment to the scaffold. Cell number observed as nuclei per field of view revealed an initial higher cell number for the conditions containing Nw. This correlated with the results obtained with Alamar Blue, where at 24 h CS-Nw presented the highest values. Over the following days, cells started to spread, displaying a stretched morphology with stress actin fibers. By the third week, a significant increase in cell density was observed, with fibroblasts forming extensive intercellular interactions and spreading across the entire scaffold. This correlates with results obtained from the proliferation studies (Figure 4B) [69].

It is important to note that the scaffolds presented some autofluorescence due to the presence of genipin. This made the investigation of cell morphology more difficult, therefore, cell morphology was also investigated via SEM imaging (Figure 5B). It was performed on scaffolds after 1 and 21 days of culture to assess potential morphological changes. At 24 h, fibroblasts appeared with round morphology as observed via fluorescence imaging [70]. In contrast, after 21 days, cells were spread, presenting multiple extensions, confirming observations from fluorescence staining.

### *In vitro* biodegradation

**Figure 6:**
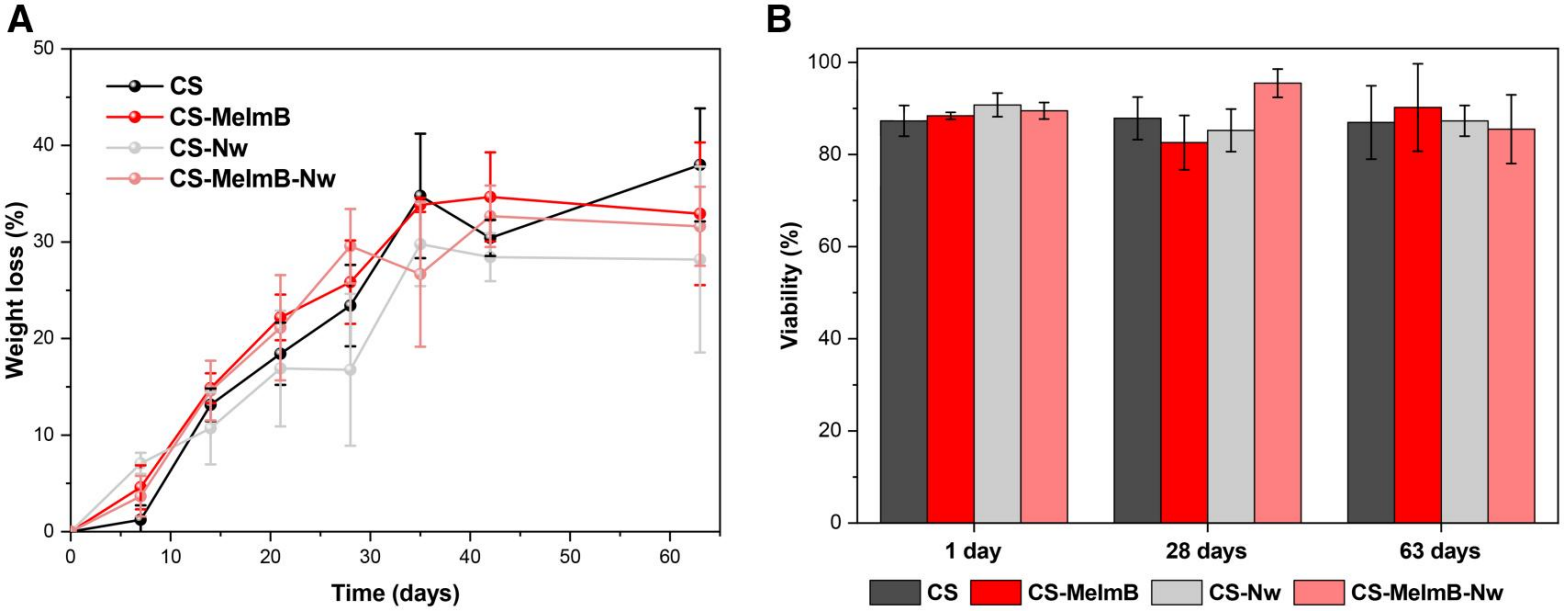
Biodegradability and by-product toxicity assessment. (**A**) Percentage of weight loss at different timepoints after treatment with lysozyme. (**B**) Percentage of cell viability after treatment with the degradation byproducts of days 1, 28 and 62 to assess their potential toxicity. s-CS (dark grey), s-CS-MeImB (dark blue), s-CS-Nw (light grey) and s-CS-MeImB-Nw (light red).

Degradability is another desirable feature in scaffolds for tissue engineering applications. The enzymatic degradation of the scaffolds was evaluated using lysozyme (Figure 6). Scaffold formulations exhibited a gradual and constant decrease in mass during the first 5 weeks. After that, approximately 30% of the initial mass was lost and the degradation curve reached a plateau. On day 63, slight differences in weight loss were observed. s-CS was the most biodegradable scaffold followed by s-CS-MeImB and s-CS-MeImB-Nw. The least degradable scaffold was s-CS-Nw. In general, the slow degradation can be attributed to the crosslinking effect of genipin, which reinforces the CS structure and reduces its susceptibility to enzymatic hydrolysis [71, 72]. The presence of Nw seemed to increase the scaffold resistance to degradation, perhaps due to the extra chitin present at the surface of the scaffold, which could have slowed down the overall degradation of the scaffold. Altogether, the structural stability provided by genipin crosslinking seemed to dominate over the influence of scaffold composition in terms of biodegradability [73].

To further assess the biological safety of the degradation byproducts, cell viability studies were conducted using fibroblasts exposed to media collected from the first, the middle and the last week of degradation (1, 28 and 63 days). Results revealed that cell viability remained above 80% in all cases, indicating that the degradation byproducts generated throughout the process were nontoxic. This supports the potential of these scaffolds for long-term tissue engineering applications, where gradual biodegradation without cytotoxic effects is essential.

### Immune response to scaffolds

**Figure 7:**
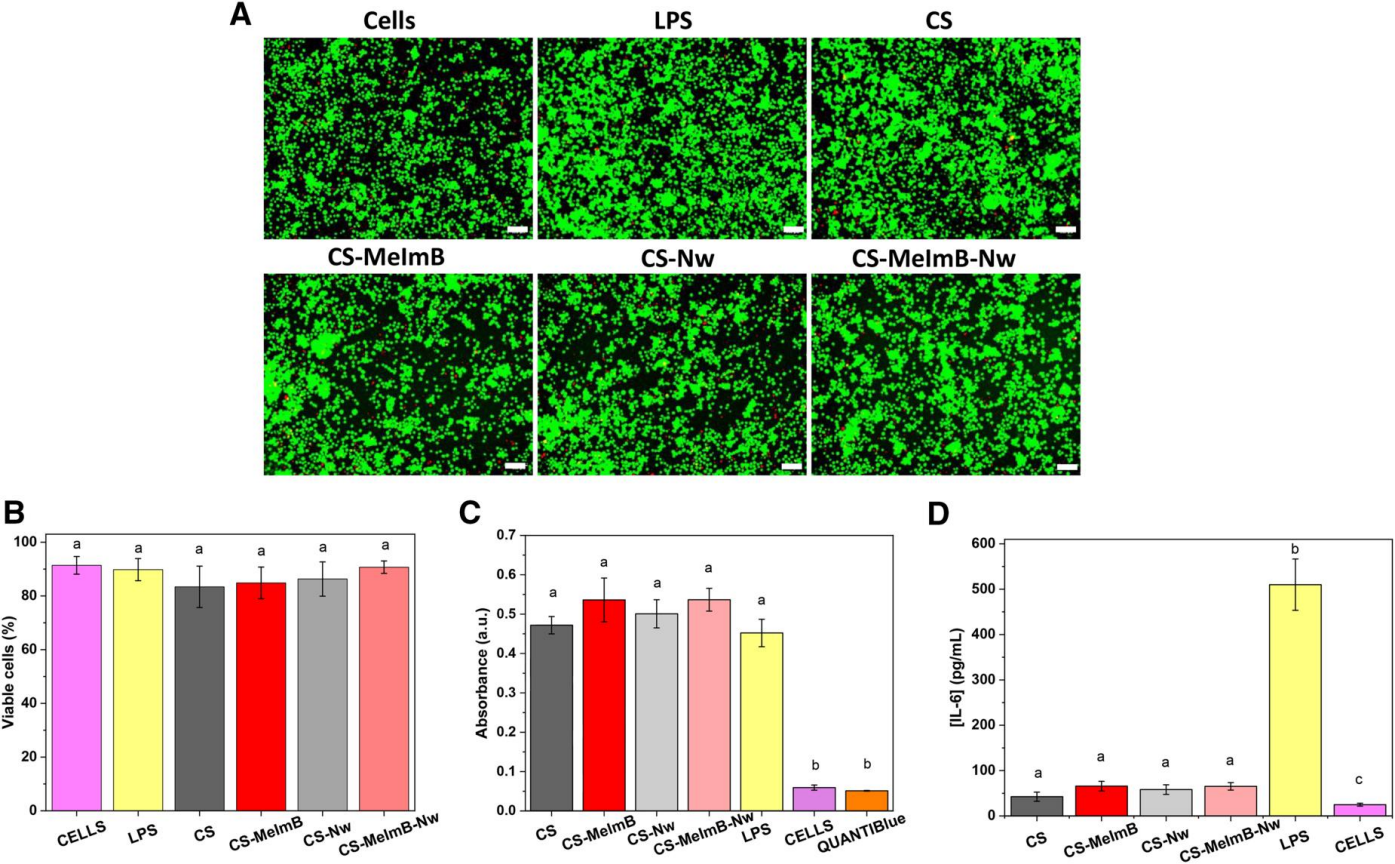
Macrophage activation potential. (**A**) Representative images of a LIVE/DEAD staining of J774.A macrophages after 24 h in contact with the scaffolds (green: alive cells, red: dead cells) (scale bar: 100 µm). (**B**) Percentage of viable cells quantified from the LIVE/DEAD staining images. (**C**) NFkB expression on macrophages after 24 h in contact with the scaffolds (positive control: treatment with LPS, negative control: cells with no treatment, blank: QuantiBlue reagent). (**D**) Quantification of IL-6 secretion from macrophages in contact with the scaffolds for 24 h (positive control: LPS treatment, negative control: cells with no treatment).

To assess the immune response triggered by the scaffolds, macrophages were cultured and put them in contact with the scaffolds for 24 h to study the potential of the scaffolds to activate the pro-inflammatory phenotype of macrophages (Figure 7). First, the viability of macrophages in contact with the scaffolds via live/dead staining and quantification of the images (Figure 7A and B) was investigated. As seen in Figure 6, the viability of macrophages in contact with the scaffolds was >90%, indicating that neither CS nor any modification was cytotoxic to the cells. This correlates with previous studies with fibroblasts, where it was observed that similar initial cell adhesion and proliferation occurred over 3 weeks of culture (Figures 4 and 5).

In order to evaluate the pro-inflammatory state of the cells, two assays were performed. First, the amount of SEAP was measured, as the cell line used is genetically modified to express SEAP when the transcription factor NFκB translocates to the nucleus [74] and, therefore, obtains an indirect measure of NFκB activation, which is a master regulator of the pro-inflammatory downstream signaling [75]. Endotoxin (lipopolysaccharide, LPS) as a positive control was used, as it is a well-known pro-inflammatory molecule [76]. Then, the amount of IL-6 secreted by the cells, another key cytokine during the inflammatory cascade was quantified [77]. Results are shown in Figure 7C and D.

NFκB activation was similar for all scaffolds investigated and similar to levels obtained for the positive control (LPS). However, the quantification of IL-6 secretion showed that cells in contact with the scaffolds did not secrete high amounts of IL-6 compared to the positive control, for which secretion of IL-6 was significantly high. The amounts of IL-6 secreted for the conditions in contact with the scaffold were more similar to the negative controls (cells without scaffold or LPS treatment). This suggests that even though macrophages seem to have NFκB activation, the downstream signaling is not observed, in this case, the production of IL-6. It is known that CS scaffolds can influence the metabolic activity of macrophages and the secretion of pro-inflammatory cytokines, which could explain the elevated NFκB levels observed [78]. This state might be reversible and perhaps longer cultures could reveal if there is a full activation cascade triggered by the presence of the scaffold or just an initial stress that could be reversed.

## Conclusions

In this study, novel s-CS-MeImB scaffolds crosslinked with genipin were developed. The resulting structure of the matrix exhibited small but highly interconnected pores, promoting fibroblast adhesion and proliferation, as confirmed by AlamarBlue assays and microscopy. At first, fibroblasts presented a rounded morphology but progressively spread and colonized the scaffolds over time. The incorporation of CS-MeImB introduced significant modifications to the chemical properties of the scaffold due to increased polarity, which influenced pore interconnectivity, water uptake, protein adsorption and biological interactions. Plus, it conferred scaffolds with antioxidant and antimicrobial activities. However, the inclusion of chitin nanowhiskers did not reinforce the rheological behavior of the scaffold architecture. The scaffolds were biodegradable up to 30% weight loss, showcasing the effect of genipin crosslinking in the stability of the scaffolds. Overall, none of the scaffolds were cytotoxic (to fibroblasts or macrophages) and the degradation byproducts were nontoxic. Immune activation studies with macrophages revealed an initial NFκB activation; however, the critical cytokine IL-6 production was not observed. This suggests that while the scaffold interacts with immune cells, it does not trigger excessive pro-inflammatory cytokine release.

In conclusion, these findings show the great potential of the MeImB-modified CS scaffolds for soft tissue engineering applications such as skin and cartilage regeneration, as they combine enhanced mechanical properties, stability, a structured porous matrix and favorable cell interactions while maintaining a controlled immune response.
